# Recognized focused practice: Does sub-specialty designation offer value to the neurosurgeon?

**DOI:** 10.1371/journal.pone.0189105

**Published:** 2017-12-14

**Authors:** Maya A. Babu, Linda M. Liau, Fredric B. Meyer

**Affiliations:** 1 Department of Neurological Surgery, Jackson Memorial Hospital, Ryder Trauma Center, University of Miami, Miami, Florida, United States of America; 2 Department of Neurosurgery, UCLA Medical Center, Los Angeles, CA, United States of America; 3 Department of Neurological Surgery, Mayo Clinic, Rochester, Minnesota, United States of America; Northwestern University, UNITED STATES

## Abstract

Vehicles for life-long assessment such as Maintenance of Certification tend to focus on generalist neurosurgical knowledge. However, as neurosurgeons advance in their careers, they tend to narrow their practice and increase volumes in certain specific types of operations. Failing to test the type of procedures most relevant to the practitioner is a lost opportunity to improve the knowledge and practice of the individual neurosurgeon. In this study, we assess the neurosurgical community’s appetite for designations of board-recognized Recognized Focused Practice (RFP). We administered a validated, online, confidential survey to 4,899 neurosurgeons (2,435 American Board of Neurological Surgery (ABNS) Diplomates participating in MOC, 1,440 Diplomates certified prior to 1999 (grandfathered), and 1,024 retired Diplomates). We received 1,449 responses overall (30% response rate). A plurality of respondents were in practice 11–15 years (18.5%), in private practice (40%) and participate in MOC (61%). 49% of respondents felt that a RFP designation would not be helpful. For the 30% who felt that RFP would be helpful, 61.3% felt that it would support recognition by their hospital or practice, it would motivate them to stay current on medical knowledge (53.4%), or it would help attract patients (46.4%;). The most popular suggestions for RFP were Spine (56.2%), Cerebrovascular (62.9%), Pediatrics (64.1%), and Functional/Stereotactic (52%). A plurality of neurosurgeons (35.7%) felt that RFP should recognize neurosurgeons with accredited and non-accredited fellowship experience and sub-specialty experience. Ultimately, Recognized Focused Practice may provide value to individual neurosurgeons, but the neurosurgical community shows tepid interest for pursuing this designation.

## Introduction

The lay press and the public have been more attentive in recent years to continuing medical education for practicing surgeons and the safeguards that exist to ensure that practitioners are delivering high-quality care throughout their career. The concept of Maintenance of Certification (MOC), namely, testing throughout a physician’s career to assure competence and safety, was developed under the auspices of ensuring that surgeons remain cognizant of the latest evidence-based practice. MOC has been institutionalized across specialties, and has historically been a general fund of knowledge of assessment. However, as a surgeon’s career progresses, it is not uncommon to develop a “focused practice” with a niche within one of the surgical sub-specialties (i.e. spine or skull base in neurosurgery). To subject all practitioners to a general fund of knowledge exam which may not represent his or her active clinical practice seems to miss the point for the necessity of the exam in the first place.

The American Board of Family Medicine developed a pilot program on Focused Practice designation in Hospital Medicine, given the understanding that many board-certified family physicians practice within a hospital and that this is an area of expertise that should be assessed uniquely. In 2009, the American Board of Internal Medicine also developed a pilot for Focused Practice in Hospital Medicine. Recognition of Focused Practice in Hospital Medicine (RFPHM) requires a primary certificate in Family Medicine. The American Board of Obstetrics and Gynecology is piloting Focused Practice in Pediatric and Adolescent Gynecology. On March 9th, 2017, the American Board of Medical Specialties released the process by which member boards could seek Focused Practice designation [1].

There are other methods of acknowledging practice expertise used by the American Board of Medical Specialties. A Certificate of Added Qualification (CAQ) is utilized by some member boards, and constitutes a body of knowledge or technique that is outside the traditional scope of the specialty practice. For instance, Sports Medicine or Sleep Medicine could be assessed through a CAQ for Family Medicine.

The pre-requisites for Recognized Focused Practice in Hospital Medicine include the following: “Direct Patient Care Pathway (i.e., full-time hospital practice) with a minimum of 1,000 hospital patient encounters per year in the last 3 years, or 3,000 encounters in the last 3 years. Formal fellowship training completed in the last 3 calendar years in a Hospital Medicine Fellowship program can be counted for a maximum of 1000 patient encounters. Another pathway is the Clinical/Systems Pathway (i.e., full-time hospital medicine professional activity with a part-time hospital practice): a minimum of 250 hospital patient encounters (limited to one encounter per patient per day) per year in the last 3 years, or 750 encounters in the last 3 years. These patient encounters must comprise at least 75% of total clinical activity, and at least 50% of the remaining non-clinical professional time must be directed toward improving the care of hospitalized patients. Each individual will select the appropriate officer of the hospitals where you obtained your patient encounters to attest to your practice levels. Acceptable hospital officers would include the Division Director, Section Chief, Chief Medical Officer, Chair of Family Medicine, Service Line Chief, Medical Director, Chief Executive Officer, President, or Chair of the Board of Directors. Random audits of the attestation process via communication with the selected officer of the hospital, and enrollment in the process is the agreement to this audit process”.

In this program, practitioners must actively participate in MOC to receive recognized focused practice certification. The unique examination involved with full certification was offered first in October 2010. It includes four two-hour sections in one sitting and is valid for 10 years, with a similar fee structure to MOC.

Other specialties, including Radiology, have also explored recognized focused practice designations. The American Board of Radiology discontinued their RFP designations in cardiac CT and brachytherapy after both met with little engagement by their Diplomates.

The American Board of Neurologic Surgery has contemplated whether to offer a recognized focused practice designation in several or all of the sub-specialties (e.g., spine, skull base, etc). Before such a program could be launched, the appetite from Diplomates needed to be gauged. The present study utilized a survey of all American Board of Neurologic Surgery diplomates as to their views on the usefulness of a Recognized Focused Practice designation, which specialties should be included, and whether Diplomates would consider pursuing such a designation.

## Methods

A sounding board of clinicians, in concert with the Directors of the American Board of Neurological Surgery and psychometric trained staff, developed a 29 multiple-choice question and free response survey deployed through the online tool, SurveyMonkey. The survey instrument did not collect any personally identifying information and the answers were anonymous and confidential. The survey was administered to 4,899 neurosurgeons (2,435 American Board of Neurological Surgery (ABNS) Diplomates participating in MOC, 1,440 Diplomates certified prior to 1999 (grandfathered), and 1,024 retired Diplomates). Funding support was provided by the ABNS and the American Board of Medical Specialties Visiting Scholars Program. STATA software (College Station, Texas) was employed to perform descriptive analyses. The first author’s Institutional Review Board reviewed and exempted the study (16–003451). We received 1,449 responses overall (30% response rate).

## Results

31% of respondents were 50–59, 20% of respondents were 60–69, 9% were 70–79, and 4% were older than 80 (Table 1). 92% of respondents were male and 8% were female (Table 2). 18% had been in practice for 11–15 years, 18% for 16–20 years, 12% for 21–25 years, 15% for 26–30 years, and 18% for more than 35 years (Table 3). Practice location by geography was diverse with the plurality (20%) of respondents from the South Atlantic region (Table 4). Respondents represented diverse practice types including private practice (40%), academic practice (34%), hybrid practice (7%), employed practice (11%) or were retired (6%) (Table 5). 44% were ABNS certified prior to 1999 (Table 6). 61% of respondents participate in Maintenance of Certification (MOC); 40% are grandfathered and do not participate in MOC (Table 7). The majority of respondents had completed some length of fellowship training, up to two years (52%; Table 8). The majority, 62%, of fellowships were unaccredited (Table 9). When asked, 49% of respondents felt that Recognized Focused Practice designation would not be helpful (Table 10). For those who felt that an RFP designation would be helpful, many felt that it help with recognition by their hospital or practice (61.3%), it would motivate them to stay current on medical knowledge (53.4%), or it would help attract patients (46.4%; Table 11). For those not interested in pursuing a recognized focused practice designation, many cited that it would not impact their daily care of patients (66.6%), would not help their hospital or practice (58.9%), and would be another test to pay for (57.9%; Table 12). The most popular suggestions for area of Recognized Focused Practice designation were Spine (56.2%), Cerebrovascular (62.9%), Pediatrics (64.1%), and Functional/Stereotactic (52%; Table 13). The majority of neurosurgeons (35.7%) felt that a Recognized Focused Practice designation should recognize neurosurgeons with accredited fellowship experience, non-accredited fellowship experience, and sub-specialty experience (Table 14). For non-fellowship trained neurosurgeons to pursue Recognized Focused Practice, the majority of respondents believe that tracking case volumes (32.9%) should be utilized (Table 15). Of the 33.7% of respondents who stated that they would pursue a Recognized Focused Practice designation, the most popular fields were spine (42%), oncology (16.7%), and cerebrovascular (11.3%; Table 16 and Table 17).

**Table 1:** Age of respondents.

Age	Number of Respondents
30–39	60 (4%)
40–49	451 (31%)
50–59	452 (31%)
60–69	290 (20%)
70–79	136 (9%)
>80	60 (4%)

**Table 2:** Gender of respondents.

Gender	Number of Respondents
Male	1330 (92%)
Female	116 (8%)

**Table 3:** Number of years since the completion of training by respondent numbers.

Number of Years	Number of Respondents
5 years or less	48 (3%)
6–10 years	207 (14%)
11–15 years	267 (18%)
16–20 years	263 (18%)
21–25 years	180 (12%)
26–30 years	218 (15%)
>35 years	264 (18%)

**Table 4:** Respondent practice location.

Region	Number of Respondents
New England (CT, ME, MA, NH, RI, VT)	85 (6%)
Mid-Atlantic (NJ, NY, PA)	181 (13%)
East North Central (IL, IN, MI, OH, WI)	214 (15%)
West North Central (IA, KS, MN, MO, NE, ND, SD)	109 (8%)
South Atlantic (DE, FL, GA, MD, NC, SC, VA, DC, WV)	283 (20%)
East South Central (AL, KY MI, TN)	88 (6%)
West South Central (AR, LA, OK, TX)	158 (11%)
Mountain (AZ, CO, ID, MT, NV, NM, UT, WY)	89 (6%)
Pacific (AK, CA, HI, OR, WA)	210 (15%)

**Table 5:** Practice type.

Practice Type	Number of Respondents
Private Practice	575 (40%)
Academic	483 (34%)
Military	18 (1%)
Veterans Affairs	24 (2%)
Hybrid	107 (7%)
Employed by Hospital or Hospital System	153 (11%)
Retired	80 (6%)
Other	98 (7%)

**Table 6:** Time of ABNS board certification.

Time	Number of Respondents
Prior to 1999	639 (44%)
2000–2004	275 (19%)
2005–2009	252 (17%)
2010–2015	278 (19%)

**Table 7:** MOC participation.

MOC Participation	Number of Respondents
Yes	870 (61%)
No	564 (39%)

**Table 8:** Fellowship completion status.

Two-year Fellowship	One Year Fellowship	Any Fellowship Experience	No Fellowship
130 (9%)	501 (34.7%)	120 (8.3%)	695 (48%)

**Table 9:** Recognition of fellowship training.

ACGME Accredited	CAST-SNS Accredited	No	Another Accrediting Group
143 (18%)	64 (8%)	496 (62%)	94 (11.8%)

**Table 10:** “Would recognized focused practice be of value to you?”

Yes	No	No Opinion
437 (30.2%)	713 (49.3%)	296 (20.4%)

**Table 11:** If recognized focused practice would be of value to you, why?

Would help attract patients	Would help with recognition by hospital or practice	Would motivate remaining up to date on clinical knowledge	Other
224 (46.4%)	296 (61.3%)	258 (53.4%)	83 (17.2%)

**Table 12:** If recognized focused practice is not of value to you, why not?

Would not impact my daily care of patients	Would not help my hospital or practice	Would be another test to pay for	Other
591 (66.6%)	523 (58.9%)	514 (57.9%)	173 (19.5%)

**Table 13:** In what specialties should recognized focused practice be offered?

Spine	596 (56.2%)
Peripheral Nerve	368 (34.7%)
Cerebrovascular	668 (62.9%)
Critical Care	375 (35.3%)
Trauma	311 (29.3%)
Pediatrics	680 (64.1%)
Oncology	363 (34.2%)
Functional/Stereotactic	552 (52%)
Skull Base	392 (36.9%)
Pain	307 (28.9%)
Epilepsy	419 (39.5%)
Other	143 (13.5%)

**Table 14:** What do you think recognized focused practice should acknowledge?

Neurosurgeons with Accredited Fellowship	Neurosurgeons with Non-Accredited Fellowship	Non-fellowship Trained Neurosurgeons with Sub-Specialty Experience	All of the above	None of the above	No opinion
439 (31.5%)	237 (17%)	221 (15.8%)	497 (35.7%)	255 (18.3%)	222 (16%)

**Table 15:** If you think non-fellowship trained neurosurgeons should be acknowledged with a recognized focus practice designation, how should this be done? (Please select all that apply).

Tracking case volumes	Completion of an oral exam	Both	Neither	I Don’t Have an Opinion	Other
409 (32.9%)	114 (9.2%)	278 (22.4%)	193 (15.5%)	324 (26.1%)	106 (8.5%)

**Table 16:** Would you seek a recognized focused practice certification if it was offered in your area of sub-specialty focus?

Yes	No	I Don’t Know
478 (33.7%)	605 (42.7%)	334 (23.6%)

**Table 17:** If you are interested in a recognized focused practice designation, what sub-specialty would you pursue?

Pain	17 (3.4%)
Epilepsy	25 (5%)
Radiosurgery	6 (1.2%)
Functional	36 (7.3%)
Peripheral Nerve	10 (2%)
Cerebrovascular	56 (11.3%)
Critical Care	20 (4%)
Neuro-Oncology	83 (16.7%)
Spine	210 (42%)
Endovascular	14 (2.8%)
Pediatric	62 (12.5%)
Skull Base	36 (7.3%)
Trauma	36 (7.3%)

## Conclusion

Recognized focused practice is meant to mirror the day-to-day practice of the practitioner, who after training, likely has specialized in one or a handful of subspecialties. Assessment is thus supposed to be more accurate as it does not reflect areas (such as complex vascular neurosurgery) in which most clinicians would not practice. The desire to offer more relevant assessment must be tempered by not increasing the burden of test taking for those with a highly specialized practice.

Our study showed that survey respondents were fairly mixed in their enthusiasm for a Recognized Focused Practice designation. 30% felt that an RFP designation would be helpful, while 49% felt it would not, and 20% had no opinion (Table 10). For those who thought an RFP designation would be helpful, most (61%) felt it would help with recognition by their practice or hospital. For those who have completed accredited fellowship training (38%) in our study, having recognition of this additional training may provide additional value within a multi-member practice. As hospitals consolidate and combine practices, having recognition of a specific area of expertise could help a practitioner focus on the pathologies/surgeries that he or she is more interested in.

For more senior neurosurgeons, many of whom did not pursue formal fellowship training, their practice now may be highly specialized. Not having a pathway to demonstrate specific training may limit their options. Certifying bodies should not limit practice options for our most senior practitioners, so consideration of an eased structure (“grandfathering”) may allow a pathway to develop to recognize the subspecialty training of more junior practitioners, without boxing out more senior practitioners who may developed a niche practice over decades.

Those most interested in pursuing an RFP designation cited spine as the most popular area of focus. With advancements in minimally invasive techniques, and fellowships devoted to these areas, and given hospital and practitioner remuneration for procedures, it is understandable that those with specialized spine training would want this area denoted and may want to limit practitioners without this area of focus. On the other hand, senior practitioners who have had an evolving practice which now includes minimally invasive spine or other newer techniques, might argue that they should not be subjected to mandatory fellowship training, if they are trying to adopt newer techniques and keep in line with practice advancements.

Internationally, there are four national physician validation systems recognized: the American Board of Medical Specialties Maintenance of Certification Program, the Federation of State Medical Boards Maintenance of Licensure Program, the Canadian Revalidation Program and the UK Revalidation Program [2]. There have been concerns voiced by some practitioners as to the value of continuing testing, and whether career testing improves clinic practice. For instance, A study of 2601 American Board of Emergency Medicine Continuing Certification test takers found that 74% felt that their medical knowledge was reinforced, increased knowledge (67%), and made them a better clinician (40%) [3]. This was based on self-report, and not correlated to patient outcome or clinical indicators.

Development of validated, practice tailored assessment tools has been explored by other fields including Plastic Surgery [4]. For example, a six week course to teach professionalism to plastic surgery faculty and residents found that a focused curriculum was found worthwhile in teaching professionalism, leadership, and management principles [5]. Peer assisted learning has also been studied; one paper identified trusting peer relationships and found that when a nurturing environment existed, peer assisted learning could be effective in providing feedback on performance for physicians [6].

The type of practice environment a physician is in also may influence his or her affinity for maintenance of certification, and the type of educational offerings provided. For instance, a study of pediatricians enrolled in Maintenance of Certification from 2013–2014 revealed that those involved in quality improvement tended not to work for independent/private practices and were employed full time [7]. Academic or multi-specialty institutions with additional infrastructure may be better equipped to aide in the study of quality improvement necessitated by some MOC pathways.

Education in medical school and residency is being reengineered to provide a seamless continuum to independent practice. The development of the ACGME (American Council for Graduate Medical Education) Milestones and “Entrustable Professional Activities” is meant to specify skills, knowledge, and procedures that trainees should be competent with. Entrustable professional activities are observable and measurable, and are meant to map to competencies and milestones for safety and efficacy [8]. Having additional testing through life-long learning pathways such as MOC helps reinforce this knowledge, and provide updates as the evidence base changes over time.

Having more relevant assessments that mirror the practice of a practitioner has been met with support by other specialties. For example, the family physicians’ development of Performance in Practice Modules (PPMs) which helped demonstrate quality improvement participation was met with enthusiastic support within the field. 29,755 ABFM diplomates completed 38,201 PPMs and 80% stated they would change patient care after completing PPM activities. 90% endorsed a high relevance to practice [9].

Several studies suggest a correlation with higher volume of certain pathologies and procedures, and better outcomes [10–13]. Given this association, designating sub-specialty certification may drive patients to certain practitioners or centers, who in turn may develop unique expertise in treating those conditions.

Ultimately, as neurosurgical practice becomes highly sub-specialized, a formal pathway to recognize specific areas of practice may become more valuable for practitioners and patients alike. As the educational landscape changes, and with more trainees pursuing formal fellowship training, coupled with several studies suggesting procedural repetition and high volumes are correlated with better clinical outcomes, movement towards recognized focused practice designation may continue to gather momentum. At present, the appetite for offering a recognized focused practice designation to neurosurgeons is tepid.

## Supporting information

S1 File Questionnaire. (PDF)
