# Supplementary material for: Recognized focused practice: Does sub-specialty designation offer value to the neurosurgeon?
Source: PLoS One. 2017 Dec 14;12(12):e0189105. doi: 10.1371/journal.pone.0189105 (PMC5730170; doi:10.1371/journal.pone.0189105)
Supplement: S1 File — (PDF) [file pone.0189105.s001.pdf]

Diplomate Feedback on MOC

Thank you for participating in this important survey! This should only take approximately 5 – 10 minutes. Your valuable feedback will help the ABNS improve the maintenance of certification (MOC) process to better serve Diplomates.

1. How old are you?

- ◯ 30-39 years old
- ◯ 40-49 years old
- ◯ 50-59 years old
- ◯ 60-69 years old
- ◯ 70-79 years old
- ◯ >80 years old

2. What is your gender?

- ◯ Female
- ◯ Male

3. How many years has it been since you completed your training (residency or post-graduate fellowship if applicable):

- 5 years or less
- 6-10 years
- 11-15 years
- 16-20 years
- 21-25 years
- 26-30 years
- >35 years

4. Where do you practice?

- New England (Connecticut, Maine, Massachusetts, New Hampshire, Rhode Island, Vermont)
- Mid-Atlantic (New Jersey, New York, Pennsylvania)
- East North Central (Illinois, Indiana, Michigan, Ohio, Wisconsin)
- West North Central (Iowa, Kansas, Minnesota, Missouri, Nebraska, North Dakota, South Dakota)
- South Atlantic (Delaware, Florida, Georgia, Maryland, North Carolina, South Carolina, Virginia, Washington D.C., West Virginia)
- East South Central (Alabama, Kentucky, Mississippi, Tennessee)
- West South Central (Arkansas, Louisiana, Oklahoma, Texas)
- Mountain (Arizona, Colorado, Idaho, Montana, Nevada, New Mexico, Utah, Wyoming)
- Pacific (Alaska, California, Hawaii, Oregon, Washington)

5. What kind of practice are you in? (Please select all that apply):

- [ ] Private Practice
- [ ] Academic
- [ ] Military
- [ ] Veterans Affairs System
- [ ] Hybrid
- [ ] Other

Other (please specify)

6. When were you ABNS board certified?

- ◯ Prior to 1999
- ◯ 2000 - 2004
- ◯ 2005 - 2009
- ◯ 2010 - 2015

7. Do you participate in MOC?

- ◯ Yes
- ◯ No

8. If yes, which of the following did you complete on time for your last mini-cycle? (Please select all that apply):

- ☐ Part I: Professionalism and Professional Standing (MOC Cycle Application, Chief of Staff Questionnaire)
- ☐ Part II: Lifelong Learning and Self-Assessment (150 CMEs, Patient Safety Course, Self-Assessment Activity)
- ☐ Part III: Passing MOC Cognitive Exam once every 10 years
- ☐ Part IV: Improvement in Medical Practice (Key Cases, Participation in Registries, Quality Improvement Project, M&M Conference Attendance, Publication of a Clinical Manuscript)
- ☐ I do not participate in MOC

9. Have you been re-certified through MOC?

- ◯ Yes
- ◯ No

10. If you do not participate in MOC, why not?

- ◯ I'm grandfathered
- ◯ Other (please specify)

While healthcare reform has been a topic of debate for decades, concerns regarding the cost and quality of medical care have fueled public expectations for greater physician accountability and better care. The ABMS Maintenance of Certification (MOC) program was developed in response to concerns regarding healthcare value and safety. The aim of this program is to improve practice performance through a process of continuous professional development.

11. Please rate your opinion of the following statement: “Physicians should be required to participate in continuing professional improvement following initial board certification.”

- ◯ Strongly Agree
- ◯ Agree
- ◯ Neutral
- ◯ Disagree
- ◯ Strongly Disagree

12. Please rate your opinion of the following statement: “Specialty boards, working in conjunction with specialty societies, should require Diplomates to participate in programs meant to promote continuous professional development.”

- ◯ Strongly Agree
- ◯ Agree
- ◯ Neutral
- ◯ Disagree
- ◯ Strongly Disagree

13. Which of the following types of programs do you believe would represent meaningful and/or worthwhile professional development (Please select all that apply):

- ☐ Self-assessment tests
- ☐ Periodic reviews of neurosurgical case logs
- ☐ Cognitive examinations
- ☐ Quality improvement projects (example: clinical registries)
- ☐ Clinical science
- ☐ Other (please specify)

14. Please rate your opinion of the following statement: “The MOC process as currently structured provides me value as a practitioner.”

- Strongly Agree
- Agree
- Neutral
- Disagree
- Strongly Disagree

15. Have you completed a fellowship?

- Yes, a two year fellowship
- Yes, a one year fellowship
- Yes, a fellowship lasting less than one year
- No, I have not completed a fellowship

16. If yes, was your fellowship formally recognized?

- Yes, by ACGME (Accreditation Council for Graduate Medical Education)
- Yes, by CAST-SNS (Committee on Advanced Subspecialty Training-Society of Neurological Surgeons)
- No
- Yes, by other (please specify) _________

Several boards of the ABMS have developed a “Recognized Focused Practice” (RFP) designation to acknowledge additional specialized training or experience in a particular sub-specialty. While the models vary, diplomates have the opportunity to obtain an RFP designation most often after demonstrating completion of recognized sub-specialty fellowship training (either enfolded or post-graduate). The MOC process can thereafter be pursued within this particular sub-specialty in addition to taking a general MOC exam. The next several questions query the creation of such a Recognized Focused Practice designation within neurosurgery.

17. Would a Recognized Focused Practice designation within neurosurgery be of value to you?

- Yes
- No
- I don’t have an opinion

18. If yes, why? (Please select all that apply)

- [ ] Would help attract patients
- [ ] Would help with recognition by hospital or practice
- [ ] Would motivate me to remain up-to-date on my sub-specialty knowledge
- [ ] Other (please specify)

19. If no, why not? (Please select all that apply):

- [ ] Would not impact my daily care of patients
- [ ] Would not help me with my hospital or practice
- [ ] Would be another test to pay for
- [ ] Other (please specify)

20. In what sub-specialties do you think Recognized Focused Practice should be offered? (Please select all that apply):

- [ ] Spine
- [ ] Peripheral Nerve
- [ ] Cerebrovascular
- [ ] Critical Care
- [ ] Trauma
- [ ] Pediatrics
- [ ] Neurosurgical Oncology
- [ ] Functional/Stereotactic
- [ ] Skull Base
- [ ] Pain
- [ ] Epilepsy
- [ ] Other (please specify)

21. What do you think Recognized Focused Practice (RFP) should acknowledge? (Please select all that apply):

- ☐ Neurosurgeons with fellowship (ACGME certified or CAST approved) training
- ☐ Neurosurgeons with fellowship (non-accredited)
- ☐ Non-fellowship trained neurosurgeons with sub-specialty experience
- ☐ All of the above
- ☐ None of the above
- ☐ I don’t have an opinion

22. If you think non-fellowship trained neurosurgeons should be acknowledged with a Recognized Focus Practice designation, how should this be done? (Please select all that apply):

- ☐ Tracking case volumes
- ☐ Completion of an oral exam
- ☐ Both
- ☐ Neither
- ☐ I don’t have an opinion
- ☐ Other (please specify)

23. Would you seek a Recognized Focused Practice certification if it was offered in your area of sub-specialty focus?

- ○ Yes
- ○ No
- ○ I don’t know

24. If yes, what sub-specialty would you pursue?

Lately, there has been public controversy about patient safety in the context of the aging surgeon. Several medical societies including the American College of Surgeons and the American Medical Association have drafted position statements on the issue. The following questions query your perceptions of the aging surgeon.

25. Should some form of additional testing be performed for the aging neurosurgeon? (Please select all that apply):

- ☐ Cognitive testing
- ☐ Case review
- ☐ Both
- ☐ Neither
- ☐ Other (please specify)

26. Please rate your opinion of the following statement: "There should be an age-based cutoff for neurosurgical practice (including operating)."

- ○ Strongly Agree
- ○ Agree
- ○ Neutral
- ○ Disagree
- ○ Strongly Disagree

27. If so, at what age should neurosurgical practice cease?

- ○ 60 years old
- ○ 65 years old
- ○ 70 years old
- ○ 75 years old
- ○ 80 years old
- ○ 85 years old
- ○ Other (please specify)

28. Please rate your opinion of the following statement: “MOC should be tailored to accommodate the aging neurosurgeon.”

- ◯ Strongly Agree
- ◯ Agree
- ◯ Neutral
- ◯ Disagree
- ◯ Strongly Disagree

29. For neurosurgeons 65 and older, MOC exams should reflect (Please select all that apply):

- ☐ Daily practice patterns via case-based testing
- ☐ Traditional question-and-answer format
- ☐ Review of individual case logs and patient outcomes
- ☐ Other (please specify)

30. Please provide any comments you may have on any aspect of the MOC process:
